# Strategies based on *nido*-carborane embedded indole fluorescent polymers: their synthesis, spectral properties and cell imaging studies

**DOI:** 10.3389/fchem.2024.1389694

**Published:** 2024-08-01

**Authors:** Lei Wang, Lingwei Mao, Xibing Feng, Shuo Wang, Guofan Jin

**Affiliations:** ^1^ Affiliated People’s Hospital of Jiangsu University, Zhenjiang, China; ^2^ School of Pharmacy, Jiangsu University, Zhenjiang, China

**Keywords:** acrylic resin, nido-carborane, indole fluorescent, eyeball, cell imaging

## Abstract

The continuous preparation scheme EPO-Poly-indol-*nido*-carborane (E-P-INDOLCAB), L100-55-Poly-indol-*nido*-carborane (L-P-INDOLCAB), RS-Poly-indol-*nido*-carborane (S-P-INDOLCAB), and RL-Poly-indol-*nido*-carborane (R-P-INDOLCAB) were used to prepare the four types of acrylic resin-coated nido-carborane indole fluorescent polymers. After testing their spectral properties and the fluorescence stability curve trend at various acidic pH values (3.4 and 5.5, respectively), L-P-INDOLCAB and S-P-INDOLCAB were determined to be the best polymers. The stable states of the two polymers and the dispersion of the nanoparticles on the system’s surface during Atomic Force Microscope (AFM) test are shown by the zeta potentials of −23 and −42 mV. The dispersion of nanoparticles on the system’s surface and the stable condition of the two polymers were examined using zeta potential and atomic force microscopy (AFM). Transmission electron microscopy (TEM) can also confirm these findings, showing that the acrylic resin securely encases the interior to form an eyeball. Both polymers’ biocompatibility with HELA cells was enhanced in cell imaging, closely enclosing the target cells. The two complexes displayed strong inhibitory effects on PC-3 and HeLa cells when the concentration was 20 ug/mL, as validated by subsequent cell proliferation toxicity studies.

## Introduction

**FIGURE 1:**
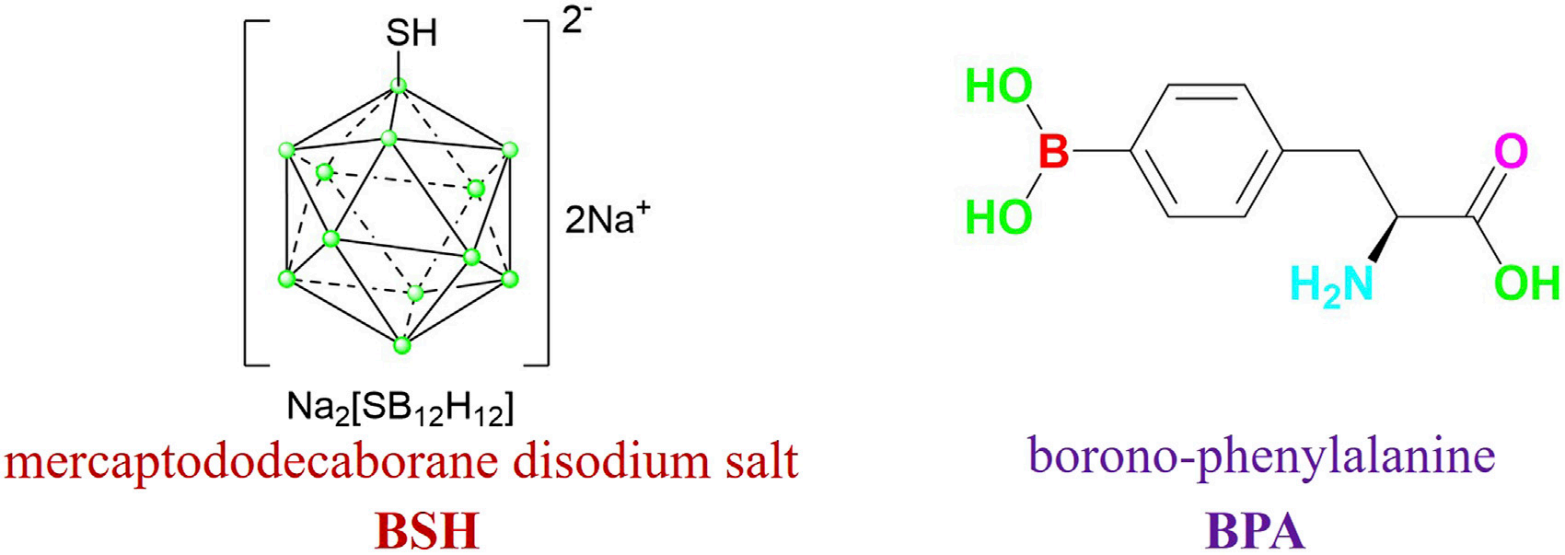
BSH and BPA.

As the first choice of anti-tumor targeted drugs in the field of boron neutron capture therapy (BNCT), carborane has been recognized by many scholars and medical institutions for its excellent tumor killing effect, high selectivity and high aggregation of tumor cells (Bai et al., 2023; Beerwerth and B¨ohmer et al., 2023). Two BNCT medicines that have been clinically utilized are mercaptododecaborane disodium salt (BSH) and borono-phenylalanine (BPA) (Figure 1). Whereas BPA targets skin cancer from melanoma, BSH targets brain gliomas (Ecer et al., 2023). Despite the two medications’ clinical recognition, many resistant tumor fields will not be able to afford them due to their poor selectivity, high cost, and low ability to agglomerate target cells. Furthermore, the solubility of water is one of the first issues to be resolved due to its potent fat-soluble impact, particularly in terms of target cell biocompatibility.

One of the most extensively used materials is acrylic resin, which is utilized in energy, textiles, and medicine. It is essentially a polymer made up of several cross-linking networks that combine methyl methacrylate, methyl acrylate, n-butyl acrylate, n-butyl methacrylate, and ethyl acrylate (Hasani et al., 2021; Hayakawa et al., 2023). They release the medication to its maximum potency concentration gradually as they break down in the stomach’s acidic environment (Hu et al., 2022; Lee et al., 2023; Lu et al., 2023; Mohamed et al., 2023). Additionally, acrylic resin is the most commonly utilized substance for drug coatings since it is non-toxic, has a high degree of stability, and is simple to metabolize and release from the body. Additionally, its makeup may be separated into two categories: oil-in-water and oil-in-water double liposome composite materials, which are also used in the cosmetics and medical industries (Nagata et al., 2023; Pariary et al., 2023).

Our design approach is to start from the visual carborane point of the view: Firstly, solve the water solubility of carborane to make it biocompatible with tumor cells (Shao et al., 2021; Phung et al., 2023; Sforzi et al., 2023); Second, because fluorophore, also known as indole quaternary ammonium salt, is extensively employed in several biological imaging (Shao et al., 2022; Shi et al., 2023; Valibeknejad et al., 2023). For tumor staining, for instance, three types of ring staining can be used: complete, partial, and marginal. Furthermore, it can persist for a very long time in the tumor environment (Wang et al., 2021; Xu et al., 2021; Wang et al., 2023). Furthermore, a tiny amount of long-acting fluorescence can also have an effect on the label. Therefore, these spectrum features may be employed to join with the *nido*-carborane potassium salt in the form of ionic bond to make it have strong fluorescence effect (Zhao et al., 2021; Zhang et al., 2023; Zimina et al., 2023). Finally, the indole-*nido*-carborane complexes were progressively coated with the characteristics of different acrylic resins for various spectrum, release, imaging and bioactivity assessment.

## Experiment

### Materials and instruments

The reagents and solvents used in this study were commercially purchased without further purification. Absorption spectra were recorded by UV-2550 spectrophotometer. Fluorescence emission spectra were recorded by Shimadzu RF-5301PCS. TEM was taken by HT7800 120 KV transmission electron microscope. Zeta potential was collected on Zetasizer Nano ZS90. All optical measurements were performed at room temperature.

### Synthesis of Poly-indole

Indole polymer (100 mg), 1,4-phthalaldehyde (20 mg) and 0.5 drop piperidine were dissolved in EtOH (5 mL) at 80°C stirred for 2 h. Without purification and next step.

### Synthesis of Poly-indole-*nido*-carborane

*nido*-carborane (20 mg) and **Poly-indole** (100 mg) were dissolved in EtOH (5 mL) at 50°C stirred for 2 h. Without purification and next step.

### Preparation of E-P-INDOCAB, L-P-INDOCAB, R-P-INDOCAB and S-P-INDOCAB

P-INDOCAB (120 mg) dissolved in ethanol (3 mL) was coated with four different acrylic resins E-P-INDOCAB, L-P-INDOCAB, R-P-INDOCAB and S-P-INDOCAB at 50°C for 2 h, respectively.

### Cellular uptake and localization by transmission electron microscope

The transmission electron microscope (TEM) was a Zeiss Ultra Plus operating at 15 keV accelerating voltage coupled to an Oxford Instruments X-Max 60 mm^2^ SDD X-ray microanalysis system. The sample’s ethanol-suspended precipitate was then added to a silicon wafer, and conductive glue was used to secure the sample to a sample tray. Following that, TEM images were taken at 2.0 m and 200 nm rulers, respectively, using a scanning electron microscope. The copper net is first covered with a thin backing sheet, and then the proper quantity of powder and tetrahydrofuran are added to the small beaker. After that, ultrasonic oscillation is done for ten to 30 minutes. After three to 5 minutes, the uniform mixture of powder and tetrahydrofuran is drawn through a glass capillary tube. Two to three droplets of the mixture are then placed onto the copper net and allowed to dry. Tetrahydrofuran should be allowed to volatilize for a minimum of 15 minutes. Finally, arrange the sample on the sample table and insert it into the electron microscope for analysis.

### Zeta potential test

A portion of the sample was removed, and after adding a little amount of ethanol, it was sonicated for 5 min. To scatter the sample, an equal volume of deionized water is added, sonicated, and shaken for 20 min. The next step is to measure each sample three times using a zeta potential analyzer.

### *In vitro* release test

After making phosphate buffers with pH values of 3.0, 4.0, and 5.0, each buffer was mixed with 5% sodium dodecyl sulfate (SDS, w/v). Plotting the absorbance standard curve of sample concentration was done after the absorbance was measured at 650 nm. To create a 2.0 mg/mL solution, the appropriate amount of the material to be analyzed was dissolved in the three types of surfactant mentioned above that contained phosphate buffer. The dialysis bag (MD34-14000) was filled with 2.0 mL of the drug-carrying micellar solution (equivalent to 2.0 mg/mL). The dialysis bag was then submerged in 20 mL of release medium. The trials on release were conducted at 37°C with a continuous temperature oscillation of 100 rpm/min. At 0.5, 1, 1.5, 2, 3, 4, 5, 6, 8, 10, 12, 16, 20, 24, 36, and 48 h, samples were collected. A new 3 mL of buffer was added to the 3 mL sample volume. The absorbance of the release medium at 650 nm was measured at each time point, and the content of L-P-INDOCAB and S-P-INDOCAB in the release medium was calculated according to the standard curve, and then the cumulative release amount of L-P-INDOCAB and S-P-INDOCAB were calculated according to the following formula.


$$Q\left(\%\right)=\frac{C_{n}V+V_{i}\sum_{i=0}^{n-1}C_{i}}{m_{drug}}\times 100\%$$ (1)

Q: Percentage of cumulative drug release, %.

Cn: Concentration of the *n*th sample taken, μg/mL.

V: Total volume of release medium, 20 mL.

Vi: Sampling volume at time point i, 3 mL.

Ci: Concentration of the sample taken at time point i, μg/mL.

m: Quality of S-P-INDOLCAB/L-P-INDOLCAB in drug-loaded micelles, μg.

### Cell imaging

HeLa cells in the logarithmic growth phase were treated with trypsin prior to seeding them on a 96-well plate with a circular cover, incubating them in an incubator with 5% CO_2_, and culture them for 24 h at 37°C to encourage adhesion. The produced polymer L-P-INDOLCAB and S-P-INDOLCAB stock solutions (20 mg/mL) were created in DMSO, respectively, and then diluted with DMSO to prepare the appropriate amounts of solution. For a full day, the cells in each sample were removed from their original growth medium and put in a medium containing 20 μg/mL. After that, it was washed twice, fixed for 25 minutes with paraformaldehyde, and disposed of using PBS. A fluorescence microscope was used to capture fluorescent pictures of the cells after the repair solution was removed with PBS and the combination was twice washed. The solution was then treated with anti-fluorescence inactivating scaffolds and incubated for 25 min in a DAPI dark room.

### Cell proliferation toxicity test (CCK8)

Trypsin digestion of PC-3 and Hela cells in the logarithmic growth phase, reorganization of the cells into a cell suspension, and modification of the cell suspension’s concentration comprised the cell pretreatment process. Three wells, each containing 5000 cells injected into 96-well plates, were given to each group. Handling of cell dosage: Blank control groups were made in line with the experimental groups, and each experimental group was given a dosage of complexes at varying concentrations (0, 4, 8, 12, 16, 20, 24 ug/mL) for 24 h. After 24 h, remove the cells, add 10 L of CCK-8 solution to each well, and then leave the mixture in the incubator for an hour. Finding the absorbance value Using a microplate reader, determine the absorbance at 450 nm. Store the data, then send them in for analysis.

## Results and discussion

Several changes were initially made to the preparation process to allow the polymer to incorporate more *nido*-carboranes. The resulting intermediate is then coupled with 1,4-phthalaldehyde, and the whole polymer is closely cross-linked using benzene as a bridge to generate a multi-component quaternary ammonium bromide polymer (P-INDO) by utilizing the quaternary ammonium salt characteristics of indole to react with bromine propylene.

**FIGURE 2:**
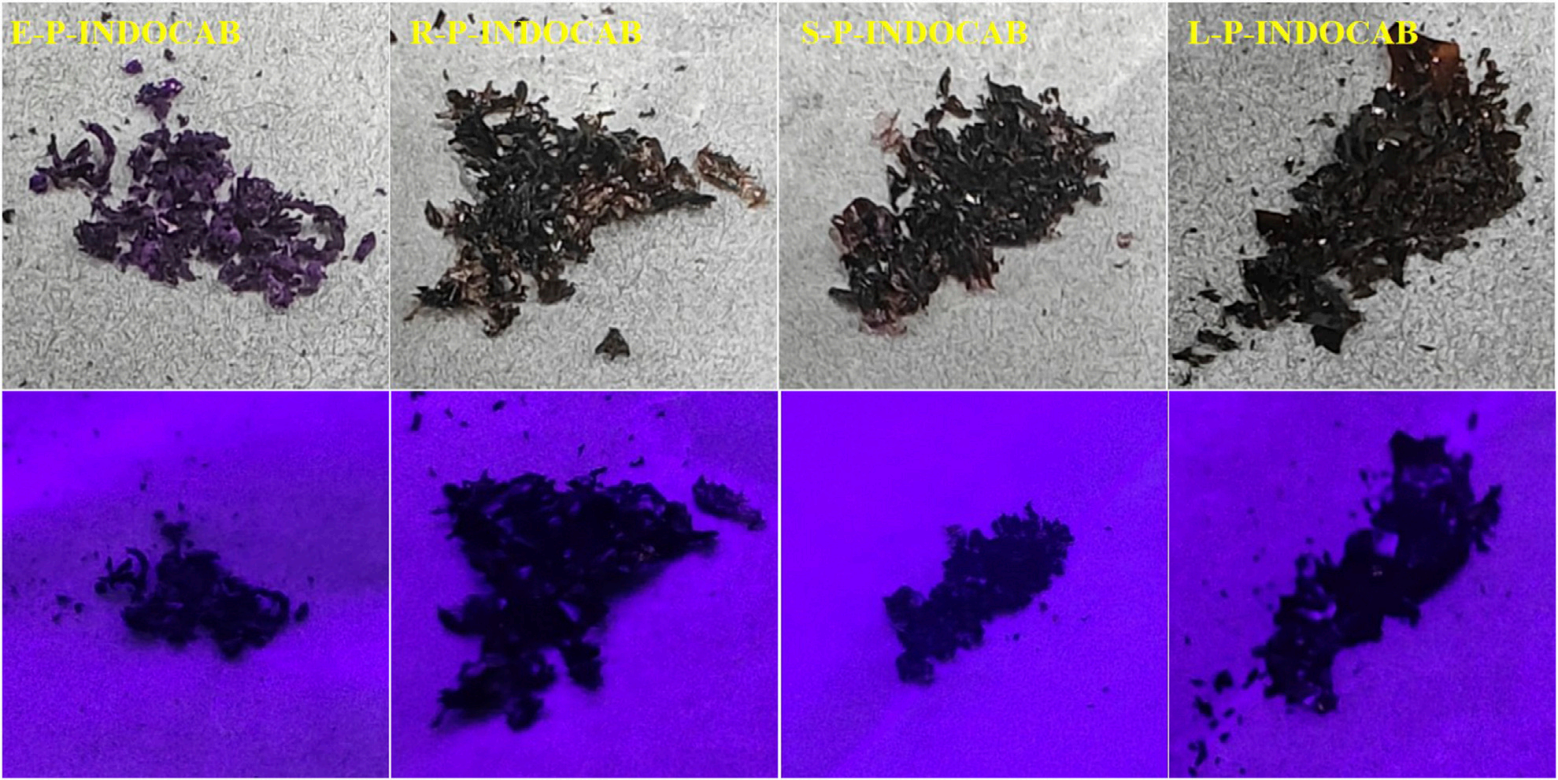
Color comparison of four polymers at sunlight and 365 nm.

**FIGURE 3:**
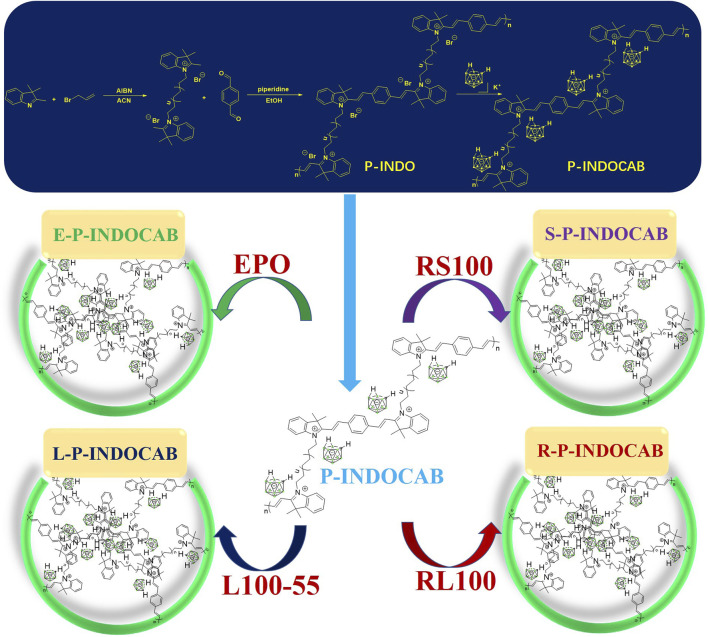
General route diagram of four kinds of indole-*nido*-carborane fluorescent polymers.

In the end, P-INDOCAB polymer with multiple *nido*-carborane was created by combining P-INDO in the form of an ionic bond with the chemical characteristics of the potassium salt based on *nido*-carborane. To allow the complex to function optimally in the gastrointestinal milieu, four distinct acrylic resin coatings were applied. Because of its structure, carborane has a noticeable impact and is highly stable, enabling delayed release and continuous activity in a variety of acidic environments (Figure 3). At 365 nm and under direct sunshine, the colors of the four produced polymers were compared. E-P-INDOCAB was dark purple in the Sun, whereas the other three polymers were purple-brown in hue. Additionally, at 365 nm, there is little variation in their colors, and they are all dark (Figure 2).

**FIGURE 4:**
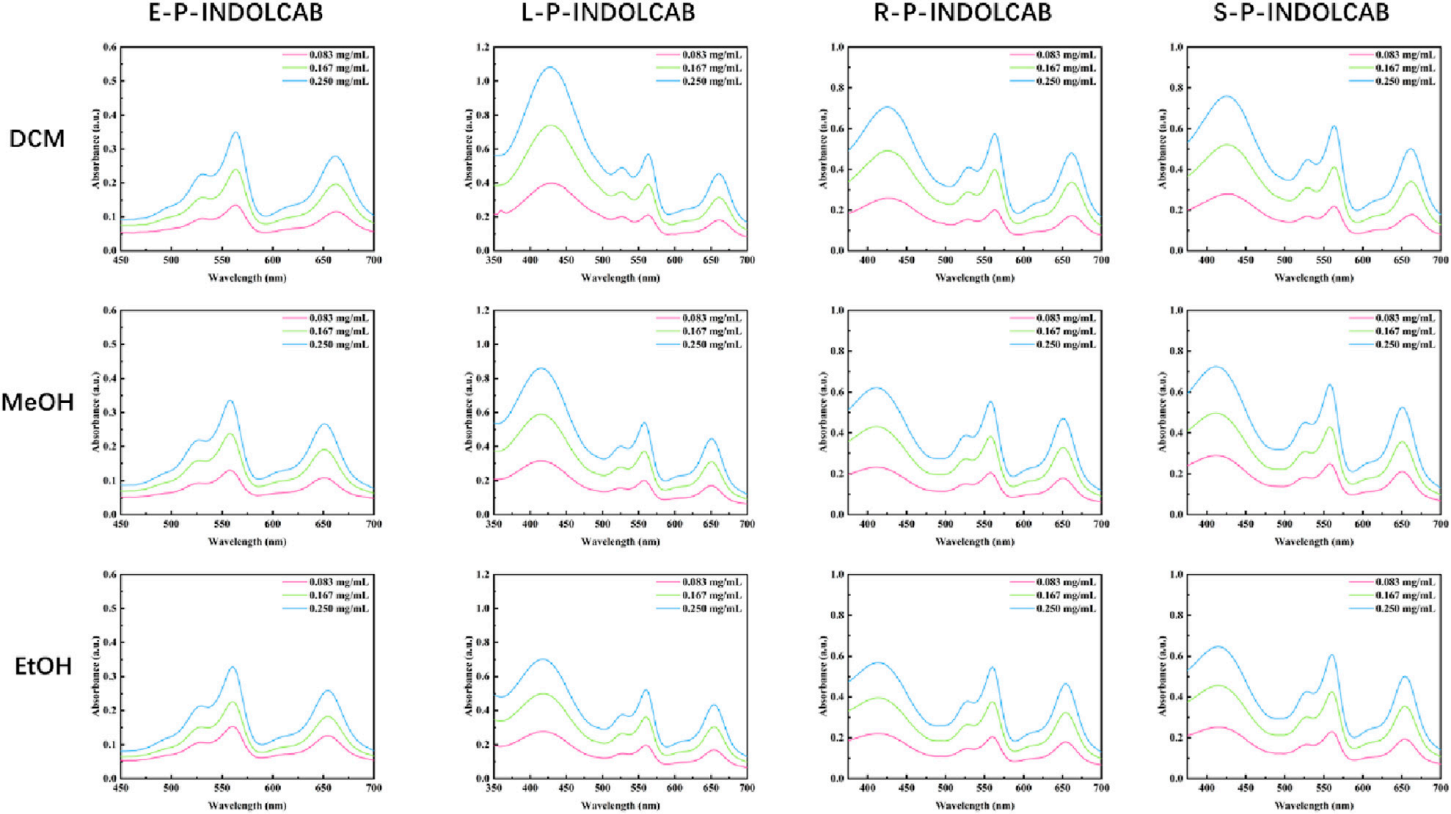
UV spectrum of four polymers in different solvents.

A number of the spectral properties of methanol, ethanol, and dichloromethane were examined in the UV spectrum. Figure 3 shows that the polymer coated with EPO exhibits two prominent absorption peaks, roughly corresponding to wavelengths of 560 nm and 650 nm. Three prominent absorption peaks may be seen at around 420 nm, 560 nm, and 650 nm for the remaining L100-55, RL100, and RS100, respectively. The occurrence of several peaks in the UV absorption peak might be attributed to the existence of different components originating from heterogeneous cross-linking of polymers during the reaction process. However, the general pattern shows that as concentration rises, so does the degree of absorption (Figure 4; Table 1)

**TABLE 1:** UV spectrum of four polymers in different solvents (0.083 mg/mL, 0.167 mg/mL, and 0.250 mg/mL).

Compound	Items	Solvents (0.083 mg/mL)		
		DCM	MeOH	EtOH
E-P-INDOLCAB	λabs/nm	563/662	558/650	560/654
L-P-INDOLCAB	λabs/nm	430/563/661	414/557/650	419/560/654
R-P-INDOLCAB	λabs/nm	425/563/663	411/558/650	413/561/654
S-P-INDOLCAB	λabs/nm	427/563/663	412/558/650	413/560/654

Compound	Items	Solvents (0.167 mg/mL)		
		DCM	MeOH	EtOH
E-P-INDOLCAB	λabs/nm	563/662	558/651	560/654
L-P-INDOLCAB	λabs/nm	429/563/661	416/558/650	417/560/654
R-P-INDOLCAB	λabs/nm	425/563/662	411/558/651	414/560/654
S-P-INDOLCAB	λabs/nm	425/563/662	411/558/651	415/560/654

Compound	Items	Solvents (0.250 mg/mL)		
		DCM	MeOH	EtOH
E-P-INDOLCAB	λabs/nm	563/661	558/651	560/654
L-P-INDOLCAB	λabs/nm	429/563/661	415/558/651	418/560/654
R-P-INDOLCAB	λabs/nm	425/563/661	411/558/651	413/560/654
S-P-INDOLCAB	λabs/nm	425/563/662	411/558/651	413/560/654

**FIGURE 5:**
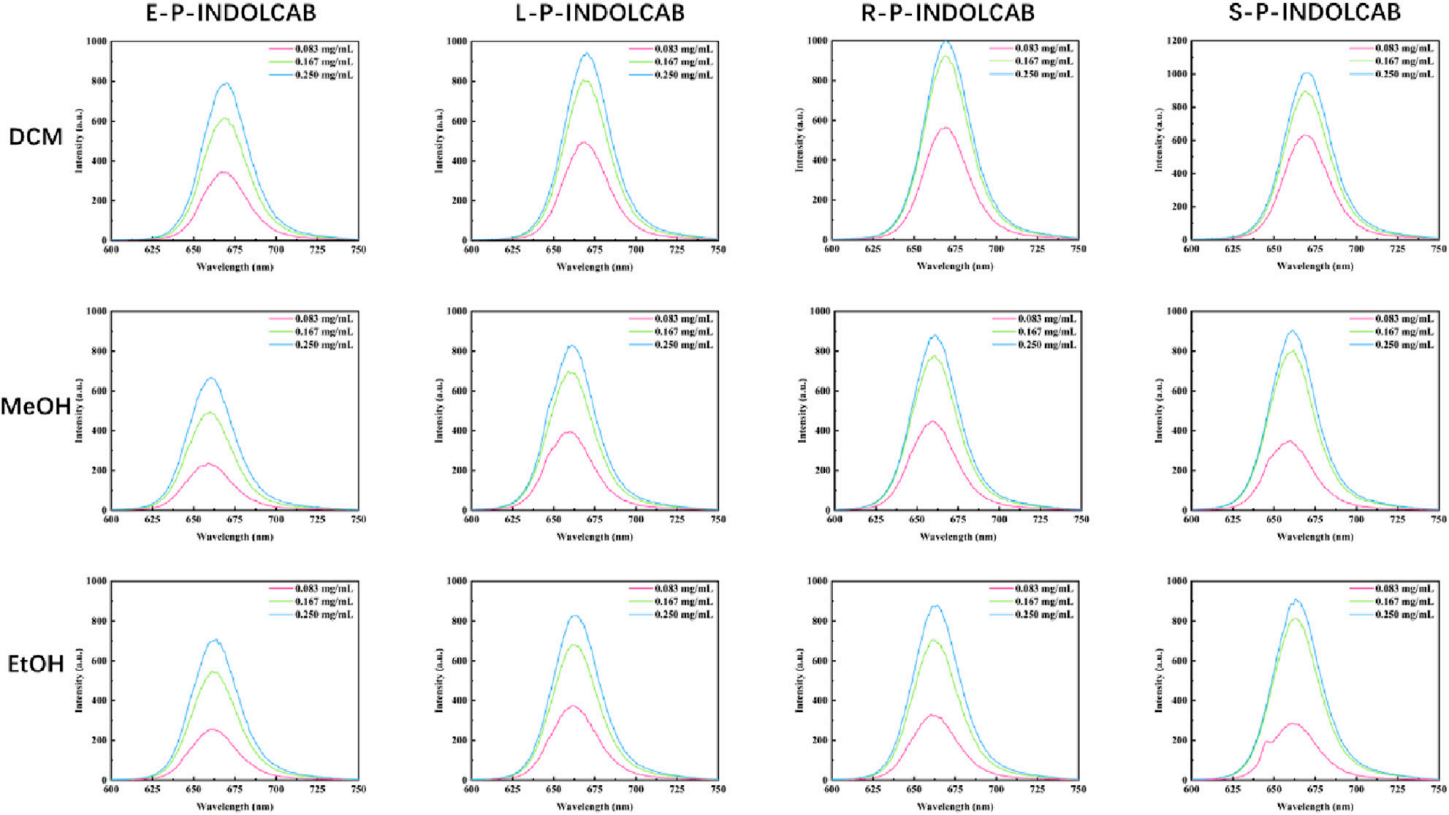
Fluorescence spectrum of four polymers in different solvents.

To observe the spectral properties of the fluorescence spectrum, the maximum value of the UV absorption data was chosen as the reference. The four polymers emit fluorescence at a wavelength of approximately 660–670 nm, and as concentration increases, so does the intensity of the emission, which increases by a factor of 4–5. This suggests that fluorescence emission intensity is more responsive to concentration than UV absorption intensity (Figure 5; Table 2).

**TABLE 2:** Fluorescence spectrum of four polymers in different solvents (0.083 mg/mL, 0.167 mg/mL, and 0.250 mg/mL).

Compound	Items	Solvents (0.083 mg/mL)		
		DCM	MeOH	EtOH
E-P-INDOLCAB	λem/nm	667	659	660
L-P-INDOLCAB	λem/nm	669	660	662
R-P-INDOLCAB	λem/nm	669	660	660
S-P-INDOLCAB	λem/nm	668	660	661

Compound	Items	Solvents (0.167 mg/mL)		
		DCM	DCM	DCM
E-P-INDOLCAB	λem/nm	669	660	661
L-P-INDOLCAB	λem/nm	668	659	662
R-P-INDOLCAB	λem/nm	669	661	661
S-P-INDOLCAB	λem/nm	669	662	663

Compound	Items	Solvents (0.250 mg/mL)		
		DCM	MeOH	EtOH
E-P-INDOLCAB	λem/nm	670	661	664
L-P-INDOLCAB	λem/nm	670	659	664
R-P-INDOLCAB	λem/nm	669	661	664
S-P-INDOLCAB	λem/nm	669	661	663

**FIGURE 6:**
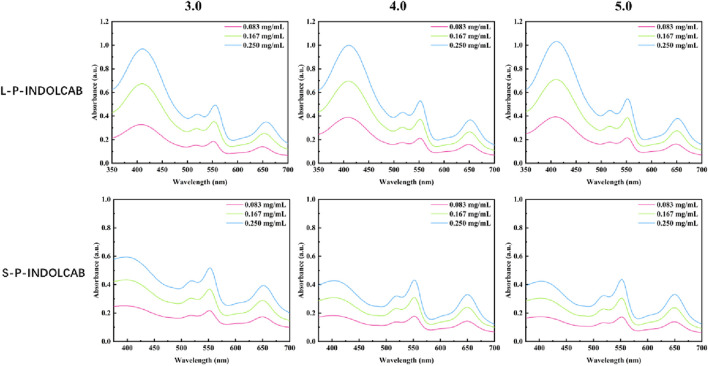
UV spectrum of four polymers under different acidic conditions.

To investigate the variations in UV absorption spectra under varying acidity conditions, the two polymers exhibiting the greatest impact were identified and subsequently contrasted. The UV absorption peak shifted to the left somewhat by around 10–15 when pH = 3.0, 4.0, and 5.0, as seen in Figure 5, while the remaining peaks essentially did not alter significantly. This demonstrates the two polymers’ excellent resilience in very acidic environments (Figure 6; Table 3).

**TABLE 3:** UV spectrum of four polymers under different acidic conditions (0.083 mg/mL, 0.167 mg/mL, and 0.250 mg/mL).

Compound	Items	Solvents (0.083 mg/mL)		
		pH = 3.0	pH = 4.0	pH = 5.0
L-P-INDOLCAB	λabs/nm	409/552/649	409/552/648	409/552/649
S-P-INDOLCAB	λabs/nm	401/552/649	399/552/649	403/552/649

Compound	Items	Solvents (0.167 mg/mL)		
		pH = 3.0	pH = 4.0	pH = 5.0
L-P-INDOLCAB	λabs/nm	411/553/653	409/552/650	410/552/650
S-P-INDOLCAB	λabs/nm	399/552/650	401/552/650	403/552/649

Compound	Items	Solvents (0.250 mg/mL)		
		pH = 3.0		pH = 3.0
L-P-INDOLCAB	λabs/nm	411/555/656	410/552/651	411/552/652
S-P-INDOLCAB	λabs/nm	398/553/652	405/552/650	403/552/650

**FIGURE 7:**
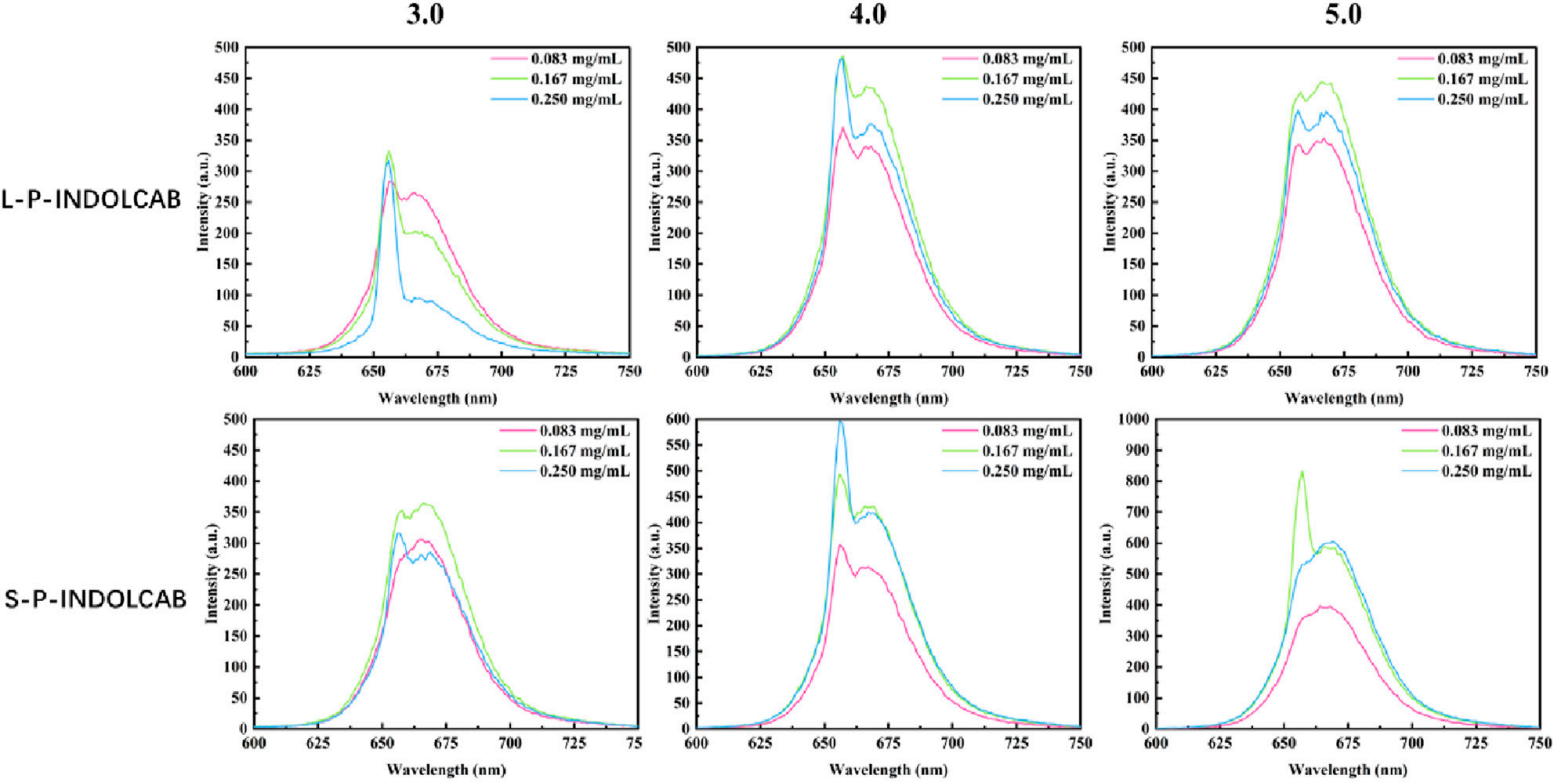
Fluorescence spectrum of four polymers under different acidic conditions.

Upon examining the variations in their fluorescence spectra at pH values of 3.0, 4.0, and 5.0, it was discovered that each polymers exhibited two distinct peaks. This is due to the fact that strong acids force the acrylic resin to start disintegrating, which causes some medications to discharge early and alters the emission spectrum’s trend. Furthermore, several curves exhibit a decline in absorption intensity as concentration increases, while the extent of these decreases remains relatively constant, indicating the excellent stability of the system (Figure 7; Table 4).

**TABLE 4:** Fluorescence spectrum of four polymers under different acidic conditions (0.083 mg/mL, 0.167 mg/mL, and 0.250 mg/mL.

Compound	Items	Solvents (0.083 mg/mL)		
		pH = 3.0	pH = 4.0	pH = 5.0
L-P-INDOLCAB	λem/nm	657/669	657/668	667/668
S-P-INDOLCAB	λem/nm	666/670	656/669	664/670

Compound	Items	Solvents (0.167 mg/mL)		
		pH = 3.0	pH = 4.0	pH = 5.0
L-P-INDOLCAB	λem/nm	656/669	657/669	666/668
S-P-INDOLCAB	λem/nm	666/669	656/670	657/670

Compound	Items	Solvents (0.250 mg/mL)		
		pH = 3.0	pH = 4.0	pH = 5.0
L-P-INDOLCAB	λem/nm	656/669	657/668	657/668
S-P-INDOLCAB	λem/nm	656/669	656/670	669/670

**FIGURE 8:**
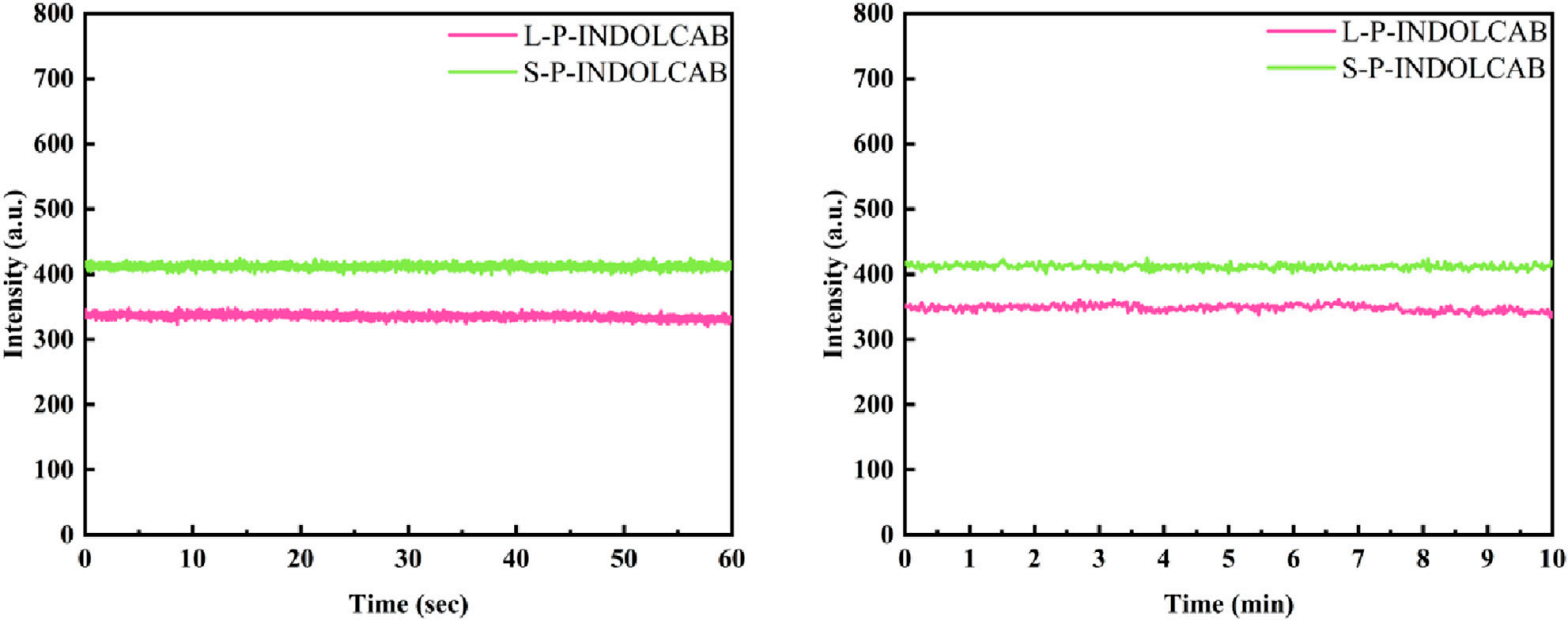
Fluorescence stability of two polymers in buffer solution for 60 s and 600 s.

In comparison to the earlier UV and fluorescence spectral data, the two polymers’ absorption and emission intensities remained relatively constant. In order to further support this perspective, the stability of the two polymers was evaluated independently in buffer solution. As shown in Figure 8, the two groups of curves are largely stable at the time period of 60 s and 600 s correspondingly, which explains their great stability in solution.

**FIGURE 9:**
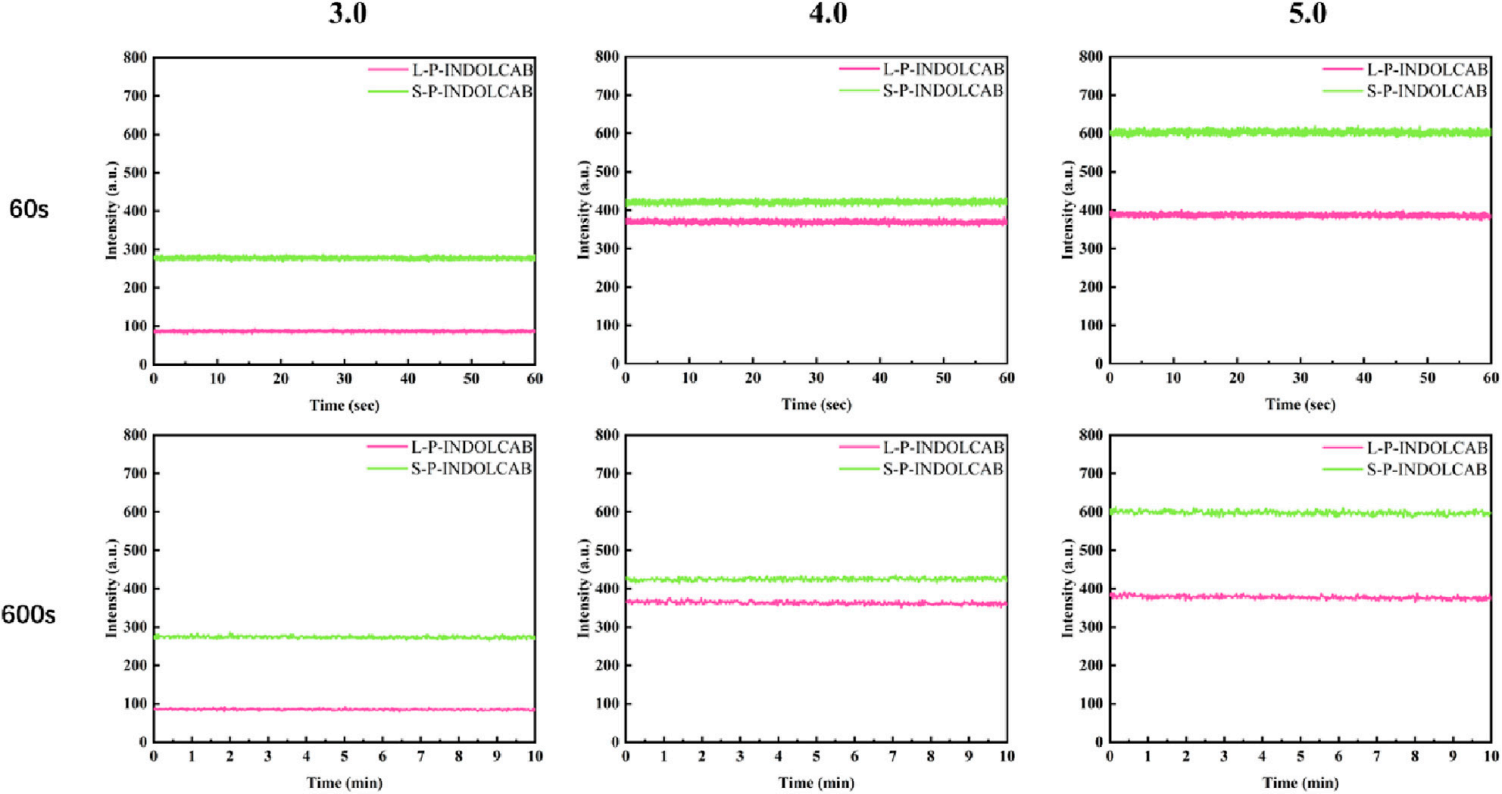
Fluorescence stability of both polymers at pH = 3.0, 4.0 and 5.0 for 60 s and 600 s.

The related findings were achieved in accordance with the aforesaid stability test in the usual buffer solution. To enhance comprehension of the stability comparison in various strongly acidic conditions, tests were conducted at 60 and 600 s for pH values of 3.0, 4.0, and 5.0. With the exception of the variation in fluorescence intensity, their curves are very smooth and stable, as shown in Figure 9.

**FIGURE 10:**
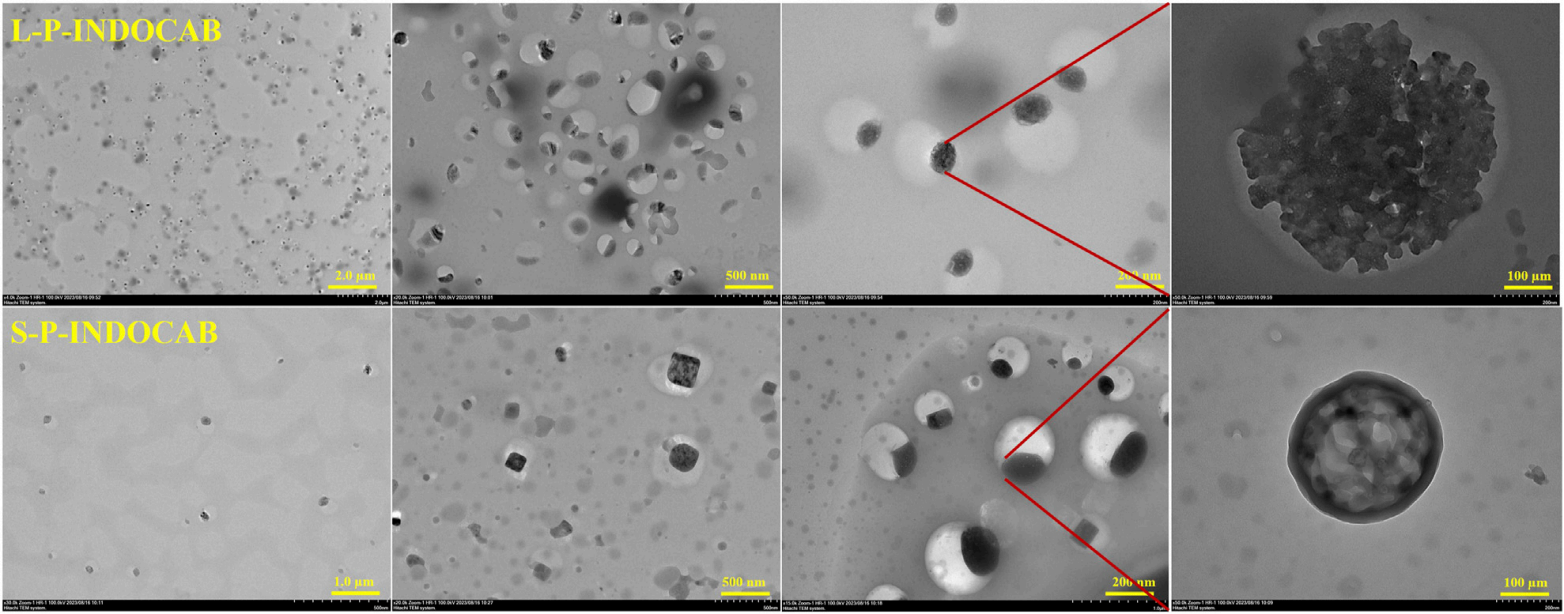
Transmission electron microscopy comparison of two polymers.

Transmission electron microscopy (TEM) was used to study the coating of L100-55 and RS100 on the indole-*nido*-carborane polymers L-P-INDOCAB and S-P-INDOCAB in order to identify the internal microscopic processes. The coating morphology of the two polymers is comparable to the eyeball, as shown in Figure 10. The black patches inside may be recognized as indole-*nido*-carborane polymer, while the white portion of the outside is composed of acrylic resin L100-55 and RS100. The outside acrylic resin securely bonds the indole-*nido*-carborane polymer to the inside. The medication’s stability and long-term release impact may both be improved by this form.

**FIGURE 11:**
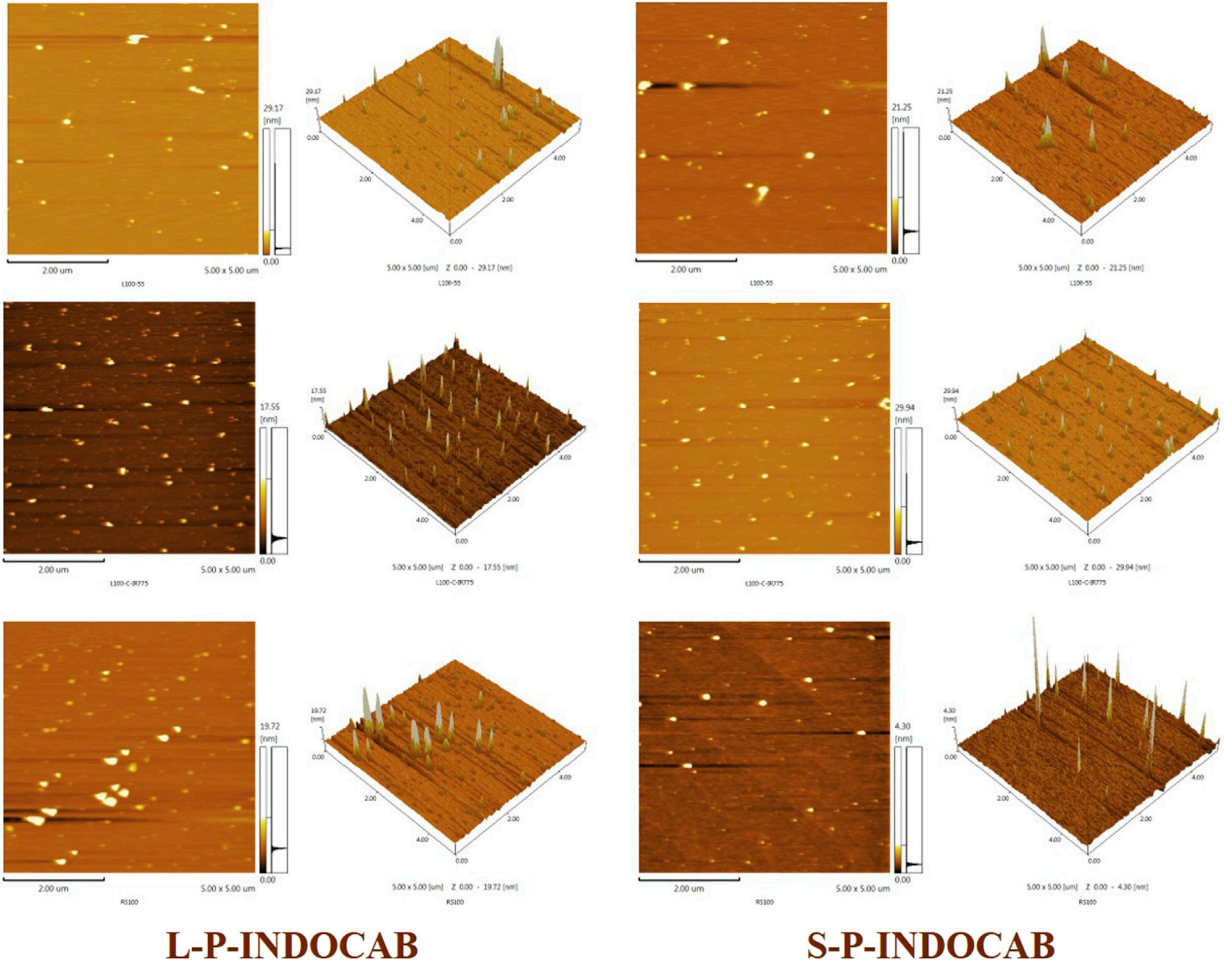
Comparison of AFM images of two polymers.

The TEM pictures that were previously reported show that the two polymers’ morphologies are almost identical. The material’s morphology was tested using an Atomic Force Microscope (AFM) to have a better look at its surface structure. It is evident from Figure 11 that the two polymers are uniformly distributed throughout the surface. Each polymer particle has a highly homogeneous size and is organized in regular three-dimensional peak columns. This AFM result is entirely in line with the earlier TEM findings.

**FIGURE 12:**
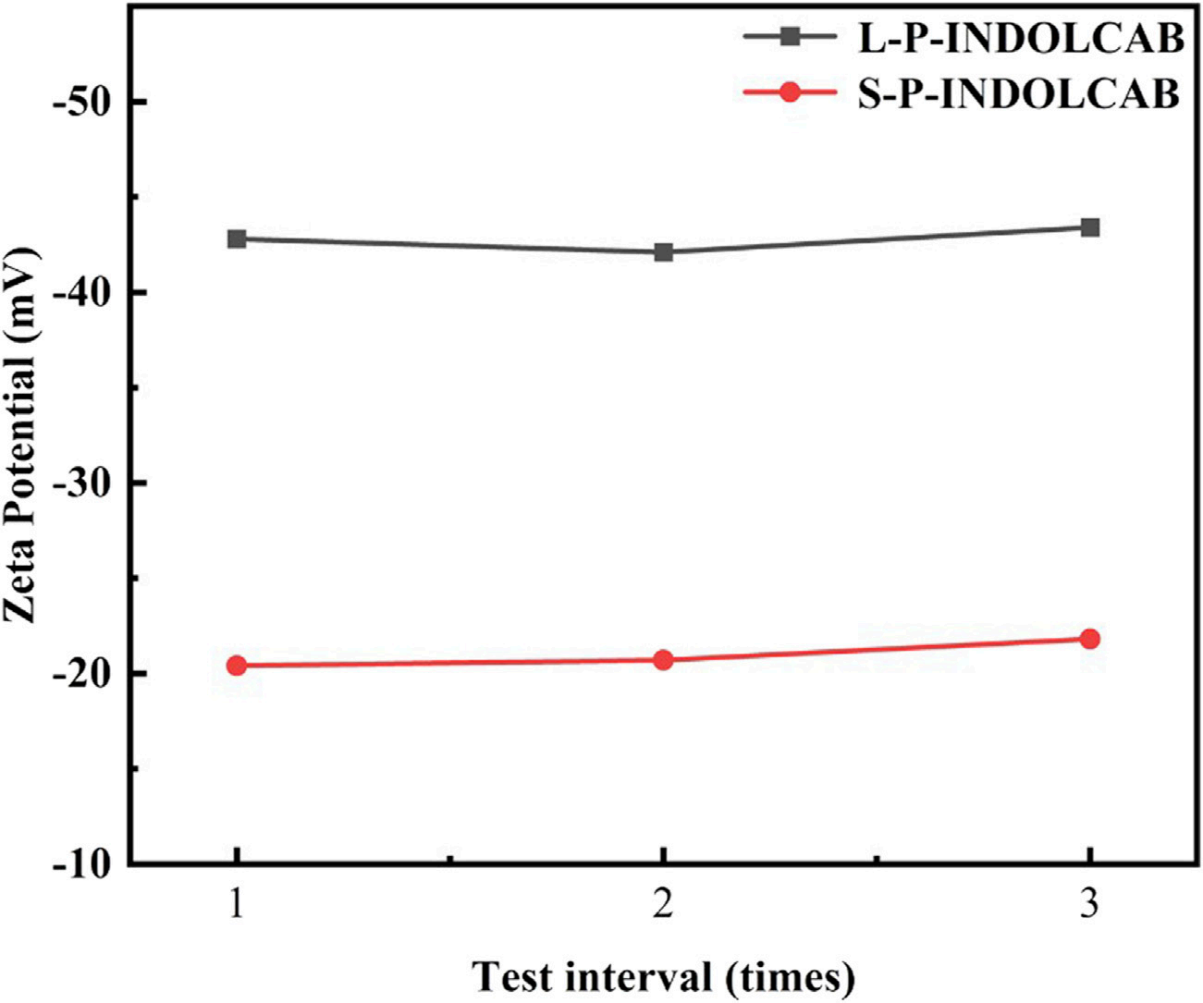
The Zeta potential values of two polymers in aqueous solution.

Zeta potential was used to measure the electronegativity of the two polymers in order to investigate their dispersion stability. The polymer potentials covered by acrylic resin L100-55 and RS100 are observed in Figure 12 to be negative, suggesting that carboxylic acid and ion forms predominate. This finding is entirely compatible with the above-mentioned experimental strategy. The difference between the two is −23 mV and −42 mV, respectively, suggesting that L-PINDOCAB covered with acrylic resin L100-55 has a somewhat greater dispersion stability than S-P-INDOCAB.

**FIGURE 13:**
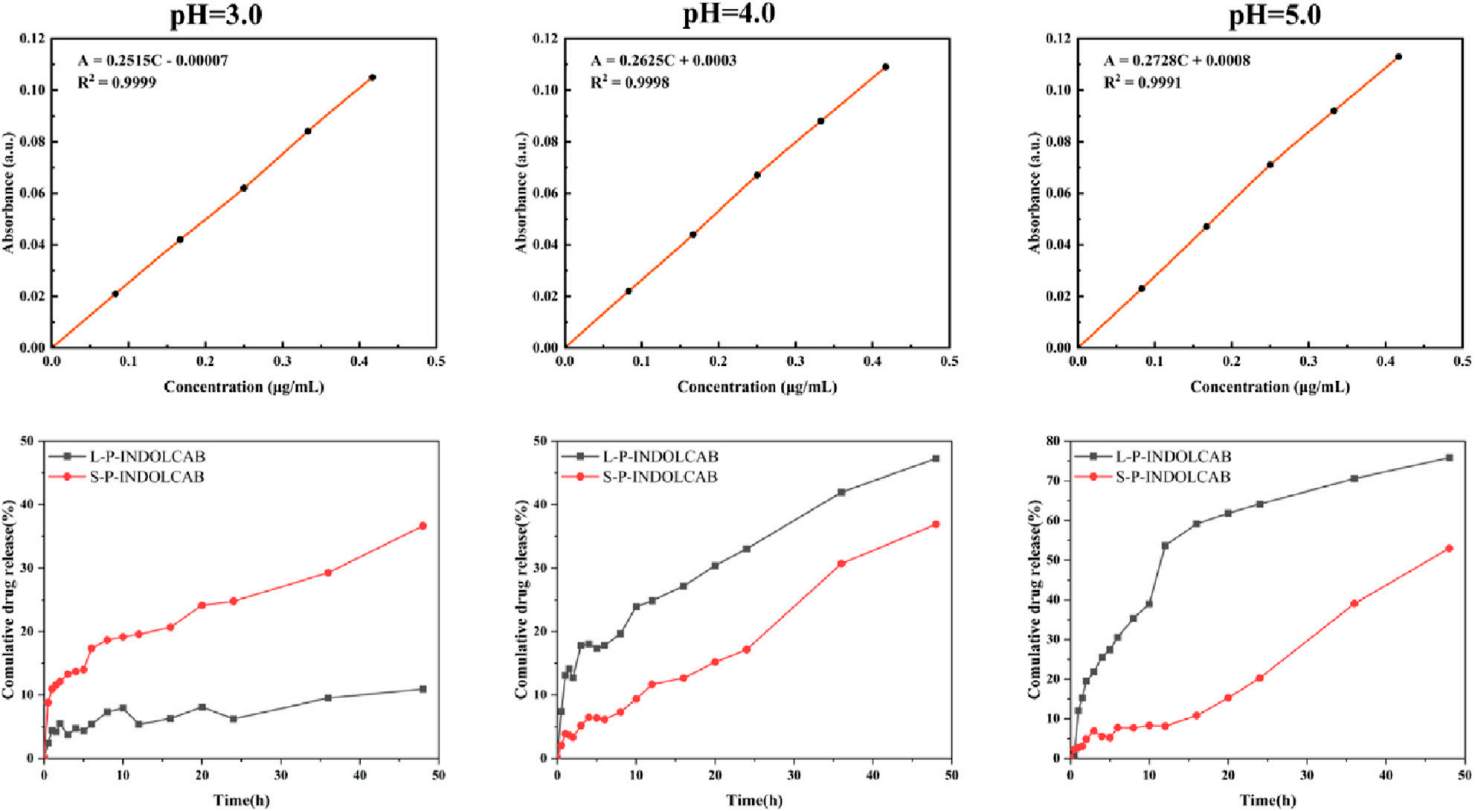
Concentration absorbance standard curve and cumulative drug release of two polymers at different pH.

In order to investigate the release efficiency of indole-*nido*-carborane polymers after packaging in the gastrointestinal environment, two polymers were released *in vitro* at three distinct pH values based on the test data mentioned above. As seen in Figure 13, the release curves for the two medications exhibit a typical phase III pattern. There are three distinct phases: the quick release stage, which lasts for 10 h; the moderate release phase, which lasts for 38–48 h; and the second, which lasts for 10–36 h. L100-55 exhibits the least quantity of drug release at pH = 3, which could be because of the charge characteristics that obstruct the drug’s transit. L100-55’s release curve is most stable around pH 5.0, and as pH rises, the overall release progressively increases. This is a result of L100-55 being a soluble resin, which makes the medication easier to release and dissolve at higher pH values. At pH = 3, RS100 released the most, whereas the releases of the other two acidic conditions were significantly less than those of L100-55. This is because RS100 is an acrylic resin that is polyionic, meaning that it dissolves and releases more readily in very acidic environments.

**FIGURE 14:**
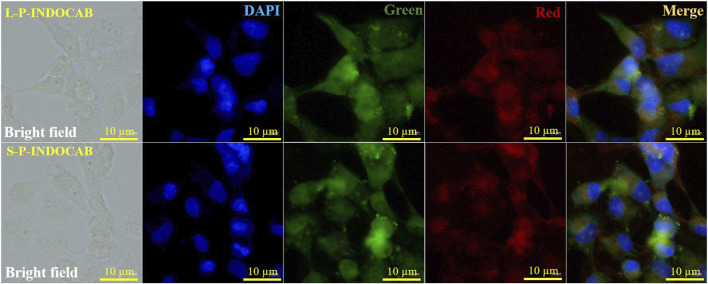
Fluorescence imaging of two polymers and HeLa cells.

Cell imaging methods were employed to investigate the biocompatibility of the two polymers in Hela cells. HeLa cells are easily visible using laser confocal microscopy and fluorescence microscopy. Figure 14 displays the green channel, red channel, merge imaging, bright field, and DAPI. The biocompatibility of both polymers was favorably received by HeLa cells. It is evident that the two green dots that are seen are polymers that adhere to and enter tumor cells. The staining impact of the green brilliant patches of tumor cells was seen in the combined and overlaid imaging, and it was also evident in the red and green channels. These findings suggest that the two polymers drive small molecule medications into tumor cells in the form of zwitterions, hence enhancing and improving the biocompatibility and aggregation of carboranes.

**FIGURE 15:**
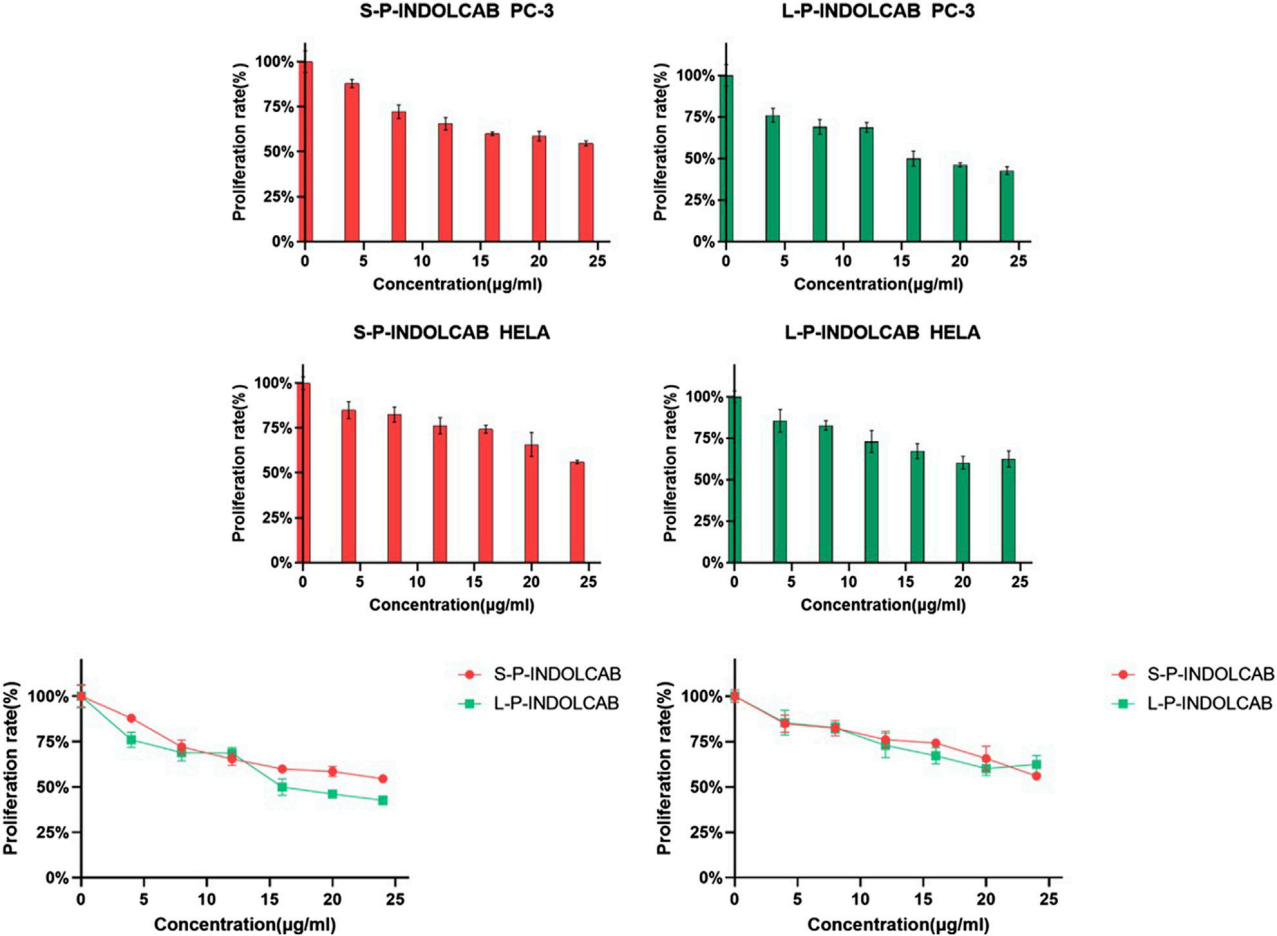
The proliferation rate of PC-3 and Hela cells treated by different concentrations of two polymers.

In order to compare the anti-proliferation effects of the two polymers on tumor cells, PC3 and Hela cells were used for the cell proliferation experiment. CCK-8 was used to measure the impact of two polymers on PC3 and HeLa proliferation. The proliferation of PC3 and HeLa cells is often strongly inhibited by these two polymers. Good biological activity was demonstrated by PC3 and HeLa treated with S-P-INDOCAB and L-P-INDOCAB, whose proliferation rates were 54.55% and 56.02%, respectively. 22.66% and 62.42% (Table 5, 14; Figure 15). Its inhibiting impact also becomes highly evident as the concentration rises. These findings demonstrated that both polymers, when coated heavily with acrylic resin, exhibited high rates of proliferation on PC3 and Hela cells.

**TABLE 5:** The proliferation rate of PC3 and Hela cells treated by different concentrations of S-PINDOCAB and L-P-INDOCAB.

Concentration (μg/mL)	0	4 (%)	8 (%)	12 (%)	16 (%)	20 (%)	24 (%)
PC3	100.00	87.90	72.14	65.45	59.95	58.58	54.55
Hela	100.00	84.94	82.45	76.18	74.25	65.74	56.02

Concentration (μg/mL)	0	4 (%)	8 (%)	12 (%)	16 (%)	20 (%)	24 (%)
PC3	100.00	76.02	68.99	68.72	49.96	46.18	42.66
Hela	100.00	85.42	82.69	72.98	67.21	60.13	62.42

## Conclusion

To sum up, the carborane fluorescent complex produced in this research is potentially an anticancer prodrug of carborane, having good biocompatibility and proliferative toxicity of malignant cells. After a strong alkali treatment, the compound of fat-soluble carborane and indole polymer was mixed in the form of zwitterion to obtain the best effect and improve its aggregation and biocompatibility to tumor cells. To create the oil-in-water form, several varieties of acrylic resins, including E-P-INDOCAB, L-P-INDOCAB, R-P-INDOCAB, and S-P-INDOCAB, were utilized as coating carriers. There is good use for the design. A novel kind of luminous polymer is produced by combining pharmacological excipients with zwitterion technology. These design ideas can offer a fundamental theoretical framework for additional study and advancement of carborane ion fluorescent polymer production.

## Data Availability

The original contributions presented in the study are included in the article/Supplementary Material, further inquiries can be directed to the corresponding author.
